# *In vivo* screening platform for shiga toxin-producing *Escherichia coli* (STEC) using *Caenorhabditis elegans* as a model

**DOI:** 10.1371/journal.pone.0193277

**Published:** 2018-02-28

**Authors:** Su-Bin Hwang, Jung-gu Choi, Shuai Wei, Byung-Jae Park, Ramachandran Chelliah, Deog-Hwan Oh

**Affiliations:** Department of Food Science and Biotechnology, College of Agriculture and Life Science, Kangwon National University, Chuncheon, Korea; East Carolina University, UNITED STATES

## Abstract

Shiga toxin-producing *Escherichia coli* (STEC) strains are the main cause of bacillary dysentery, although STEC strains generally induce milder disease symptoms compared to *Shigella* specie*s*. This study aimed to determine the virulence of STEC using the nematode *Caenorhabditis elegans* as a model host. Worm killing, fertility and bacterial colonisation assays were performed to examine the potential difference in the virulence of STEC strains compared to that of the control *E*. *coli* OP50 strains on which worms were fed. A statistically significant difference in the survival rates of *C*. *elegans* was observed in that the STEC strains caused death in 8–10 days and the *E*. *coli* OP50 strains caused death in 15 days. STEC strains severely reduced the fertility of the worms. The intestinal load of bacteria in the adult stage nematodes harbouring the *E*. *coli* OP50 strains was found to be 3.5 log CFU mL^-1^. In contrast, the STEC strains E15, E18 and E22 harboured 4.1, 4.2 and 4.7 log CFU ml^−1^ per nematode, respectively. The heat-killed STEC strains significantly increased the longevity of the worms compared to the non-heated STEC strains. In addition, PCR-based genomic profiling of shiga toxin genes, viz., stx1 and stx2, identified in selected STEC strains revealed that these toxins may be associated with the virulence of the STEC strains. This study demonstrated that *C*. *elegans* is an effective model to examine and compare the pathogenicity and virulence variation of STEC strains to that of *E*. *coli* OP50 strains.

## Introduction

Enteropathogenic *Escherichia coli* (EPEC) causes life-threatening infections in humans as a consequence of the production of shiga-like toxins. Shiga toxin-producing *Escherichia coli* (STEC) strains such as O157:H7 and non-O157 that consists of 6 serogroups, including O104, O111, O121, O145, O103 and O126, cause severe diarrhoea and haemorrhagic colitis (HC), and they can also lead to life-threatening diseases like haemolytic uremic syndrome (HUS)[1]. STEC is a pathogenic form of *E*. *coli* that causes dysentery similar to *Shigella* but with minor symptoms [2,3]. STEC is recognized as a diverse group of pathogens that closely resembles *Shigella* as it shows high similarity in specific pathogenic characteristics and certain metabolic traits [3–7]. An outbreak of diarrhoea in Germany caused by entero-aggregative STEC was associated with a high percentage of patients developing HUS. In early May 2011, this led to 782 cases of HUS (29 deaths) and 3128 non-HUS cases (17 deaths), making it the largest outbreak of HUS in the world [8]. Remarkably, the outbreak strain was serotyped as a novel O104:H4, which was not reported previously and has been associated with very few HUS cases [9]. Furthermore, there was an increase in illnesses due to non-O157 STEC strains caused by serogroups O26, O45, O103, O111, O121, and O145 as well as outbreaks attributed to STEC O26:H11, O111:H8, and O121:H19 [10]. Therefore, it is necessary that the toxic mechanisms of these bacteria be investigated further.

It is generally accepted that the presence of *Shigella* strains arose multiple times from several independent ancestral *E*. *coli* strains and that these strains are more appropriately classified as a group of pathogenic *E*. *coli* [11–14]. Enteroinvasive *E*. *coli* (EIEC), on the other hand, is thought to have evolved later than *Shigella* from different ancestral strains of *E*. *coli* [15]. In addition, further research must be done to characterize the virulent effects of STEC, which will prevent the appearance new evolved strains. Several animal models have been proposed for studying enterohemorrhagic *E*. *coli* (EHEC) infections [16–18]. However, it has been difficult to identify certain bacterial virulence factors due to the unavailability of a suitable model system to study the disease mechanism as well as numerous ethical considerations. Lack of a suitable animal model system currently hinders the development of an in vivo study of a STEC virulent strain based on systematic methods [19]. As an alternative to existing mammalian pathogenesis models, researchers have applied techniques in the study of human-pathogen interactions using *Caenorhabditis elegans* as a simple but suitable model system [20].

*C*. *elegans* is a free-living nematode and is ubiquitous in the soil environment. It is easy to culture and can be kept in a frozen state for a long duration during the hibernating stage. The steps are considered relatively simple, and it has the advantage of allowing observation of the progression from the cell to the fertilized worm. The primary food source of the nematodes in the soil environment mainly depends on bacteria, which can affect growth development, fecundity and survival [21]. The virulence mechanisms elucidated in *C*. *elegans* infected by select pathogens were found to be very similar to those in the human host, including colonisation with biofilm formation on the worms, fatal infection of the intestine, and killing by toxins [22]. Additionally, a study has been previously conducted in *C*. *elegans* on host-pathogen interactions that led to the study of the change in behavioural mechanisms. It also facilitated in distinguishing virulent and a virulent strains [23]. Therefore, *C*. *elegans* has been established as a suitable model system for the study of virulence and behaviour mechanisms of pathogenic bacteria. The intoxication and paralysis of *C*. *elegans* by strains of the EHEC serotype O157:H7 have also been documented [24–25].

Previous studies were performed on the toxicity and virulence mechanism of pathogenic strains in the nematode [26]. Studies have been conducted on the fecundity and lifespan enhancement with *C*. *elegans* as a suitable model due to its simple and short life cycle [27]. Recent studies have used the *C*. *elegans* nematode as an infection host for STEC[28–30]. *C*. *elegans* has been extensively used as a suitable, simple and reliable model to study host–pathogen interactions and identify virulence mechanisms [31–36]. In this study, we tested whether the *C*. *elegans* model can be used to distinguish virulence between toxin-producing *E*. *coli* (STEC) like O157:H7 strains and non-O157 strains, consisting of the 4 serogroups O104, O111, O121 and O145, by quantifying nematode survival rates. Further experiments demonstrated the toxic roles using the heat-killed bacterial assay, the chemotaxis assay, PCR primers for the identification of stx1 & stx2 toxin genes and the bacterial colonisation-based fertility assay as the assessing parameters (S1 Fig).

## Results

### Population density of STEC strains affects the rate of developmental progression

**Fig 1:**
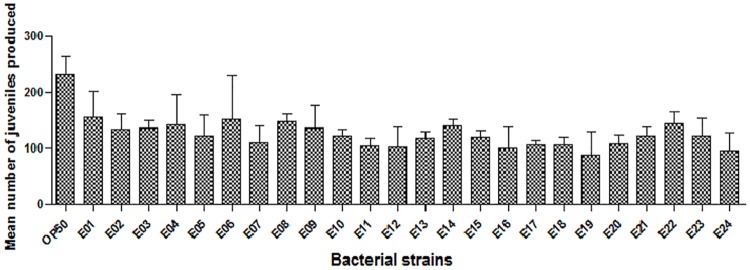
Fecundity of STEC strains in *C*. *elegans* compared to that of the *E*. *coli* OP50 strain. Brood sizes of *C*. *elegans* were examined at 20°C. n>10 in each case.

To determine whether STEC strains infect and kill *C*. *elegans* in this study concerning pathogenic and virulence mechanisms, we fed *C*. *elegans* 24 different STEC strains, which were compared to *C*. *elegans* that were given the *E*. *coli* OP50 strain as a sole food source. Single virgin hermaphrodite *C*. *elegans* were fed each STEC strain and analysed for the growth development and offspring production rate. Nematodes feeding on pathogenic *E*. *coli* (O157 and non-O157 strains) were severely infected compared to those on *E*. *coli* OP50 plates. After 4 days of exposure, the mean number of juveniles (young stage) that the *C*. *elegans* produced on the 24 STEC strains was significantly lower compared to that of the control (*E*. *coli* OP50) (Fig 1). All STEC strains reduced the reproductive capability by 30–70% compared to that of the *E*. *coli* OP50 strain. In contrast, there was no difference detected among the STEC serotypes O104, O111, O121, O145 and O157. This provides evidence that all of the 24 STEC strains used in the study decrease the size of young adult nematodes when compared to standard *E*. *coli* OP50 as the food source.

### Effect of STEC strains on *C*. *elegans* as a killing factor

**Fig 2:**
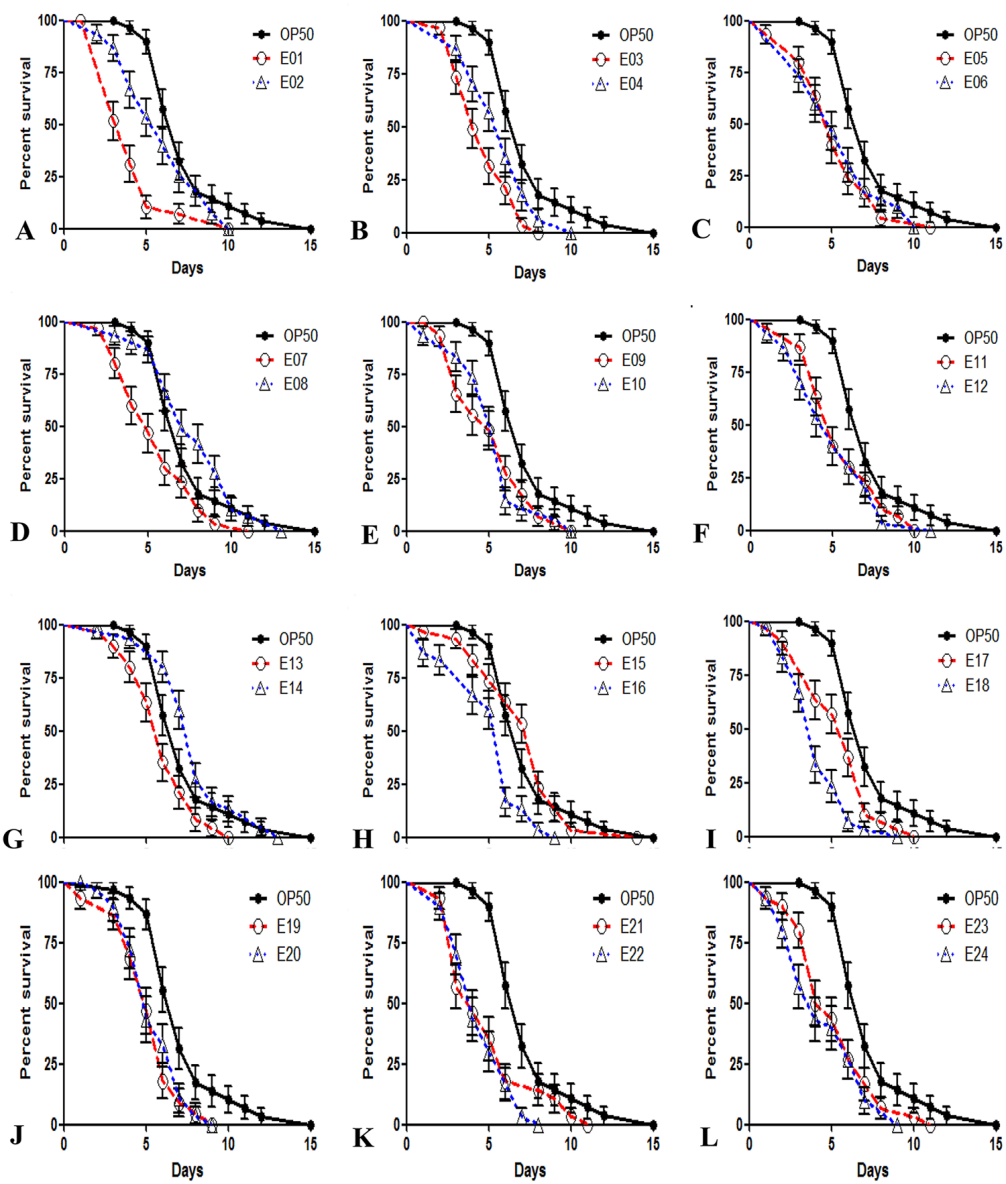
Life span assay for *C*. *elegans* infected with STEC strains compared to that for *C*. *elegans* infected with the control *E*. *coli* OP50 strain. *C*. *elegans* grown on STEC plates were significantly short-lived compared to those grown on *E*. *coli* OP50 plates. The *E*. *coli* OP50 strain was compared to 24 different STEC strains (A-L).A) Longevity of *C*. *elegans* grown on the *E*. *coli* OP50 strain compared to that of *C*. *elegans* grown on the E1 and E2 strains. *C*. *elegans* grown on both E1 andE2 exhibited a decreased life span at the end of 10 days, but *C*. *elegans* grown on *E*. *coli* OP50 survived for 15 days. B) Longevity of *C*. *elegans* grown on the *E*. *coli* OP50 strain compared to that of *C*. *elegans* grown on the E3 and E4 strains. *C*. *elegans* grown on both E3 and E4 exhibited a decreased life span, but when compared to *C*. *elegans* grown on *E*. *coli* OP50, *C*. *elegans* grown on E3 survived for 8 days, and *C*. *elegans* grown on E4 survived for 10 days. C) Longevity of *C*. *elegans* grown on the *E*. *coli* OP50 strain compared to that of *C*. *elegans* grown on the E5 and E6 strains. *C*. *elegans* grown on both E5 and E6 exhibited a decreased life span at the end of 10 days, but *C*. *elegans* grown on *E*. *coli* OP50 survived for 15 days. D) Longevity of *C*. *elegans* grown on the *E*. *coli* OP50 strain compared to that of *C*. *elegans* grown on the E7 and E8 strains. *C*. *elegans* grown on both E7 and E8 exhibited a decreased life span at the end of 10 days, but *C*. *elegans* grown on *E*. *coli* OP50 survived for 15 days. E) Longevity of *C*. *elegans* grown on the *E*. *coli* OP50 strain compared to that of *C*. *elegans* grown on the E9 and E10 strains. *C*. *elegans* grown on both E9 and E10exhibited a decreased life span at the end of 10 days, but *C*. *elegans* grown on *E*. *coli* OP50 survived for 15 days. F) Longevity of *C*. *elegans* grown on the *E*. *coli* OP50 strain compared to that of *C*. *elegans* grown on the E11 and E12 strains. *C*. *elegans* grown on both E11 and E12 showed a decreased life span at the end of 10 days, but *C*. *elegans* grown on *E*. *coli* OP50 survived for 15 days. G) Longevity of *C*. *elegans* grown on the *E*. *coli* OP50 strain compared that of *C*. *elegans* grown on the E13 and E14 strains. *C*. *elegans* grown on both E13 and E14 exhibited a decreased life span at the end of 10 days, but *C*. *elegans* grown on *E*. *coli* OP50 survived for 15 days. H) Longevity of *C*. *elegans* grown on the *E*. *coli* OP50 strain compared to that of *C*. *elegans* grown on the E15 and E16 strains. *C*. *elegans* grown on both E15 and E16 exhibited a decreased life span at the end of 9 days, but *C*. *elegans* grown on *E*. *coli* OP50 survived for 15 days. I) Longevity of *C*. *elegans* grown on the *E*. *coli* OP50 strain compared to that of *C*. *elegans* grown on the E17 and E18 strains. *C*. *elegans* grown on E17 exhibited a decreased life span at the end of 10 days, and *C*. *elegans* grown on E18 surprisingly survived for 6 days, but *C*. *elegans* grown on *E*. *coli* OP50 survived for 15 days. J) Longevity of *C*. *elegans* grown on the *E*. *coli* OP50 strain compared that of *C*. *elegans* grown on the E19 and E20 strains. *C*. *elegans* grown on both E19 and E20 exhibited a decreased life span at the end of 9 days, but *C*. *elegans* grown on *E*. *coli*OP50 survived for 15 days. K) Longevity of *C*. *elegans* grown on the *E*. *coli* OP50 strain compared to that of *C*. *elegans* grown on the E21 and E22 strains. *C*. *elegans* grown on E21 showed a decreased life span at the end of 10 days, and in contrast, *C*. *elegans* grown on E22 survived for 7 days, but *C*. *elegans* grown on *E*. *coli* OP50 survived for 15 days. L) Longevity of *C*. *elegans* grown on the *E*. *coli* OP50 strain compared to that of *C*. *elegans* grown on the E23 and E24 strains. *C*. *elegans* grown on E23 showed a decreased life span at the end of 11 days, and in contrast, *C*. *elegans* grown on E24 survived for 9 days, but *C*. *elegans* grown on *E*. *coli* OP50 survived for 15 days.

In order to determine the suitability of *C*. *elegans* as an animal model for studying STEC pathogenesis in vivo, the EHEC serotypes O104, O111, O121, O145 and O157 were tested for their ability to kill *C*. *elegans*. Nematodes were grown on a lawn of STEC strains, which were cultured on NGM plates at 20°C, and their growth development was observed for changes in body size, darkness of the intestine, and pharyngeal (mouth cavity muscles) activity based on pumping. The experiment revealed spontaneous movement in nematodes that were grown on a lawn of the avirulent *E*. *coli* OP50 strain. The nematodes grown on STEC strains were severely infected compared to those grown on control *E*. *coli* OP50 plates after 4 days, as evidenced by their pale appearance, the dilated intestinal lumen and the absence of pharyngeal pumping. As shown in Fig 2, each STEC serotype displayed a robust virulent phenotype that significantly reduced the lifespan of the nematode compared to that of the *E*. *coli*OP50 control. Nematodes feeding on *E*. *coli* O104:H12 B471 1.2673 (E1) plates displayed a median lifespan (LD_50_) of 4.24±0.43 days compared to the control median lifespan of 7.37±0.43 days for nematodes feeding on *E*. *coli* OP50 plates (Fig 2a). Nematodes feeding on *E*. *coli* O111:NM B473 96–3166 (E3) and *E*. *coli* O121:NM B483 9918 (E12) plates exhibited median lifespans of 4.75±0.29 days and 5.03±0.44 days, respectively, compared to that for nematodes feeding on the *E*. *coli* OP50 strain(Fig 2b and 2c). Also, the nematodes feeding on STEC O145:NM B489 BCL73 (E18) and *E*. *coli* O157:H7 B493 C7927(E22) plates appeared to have median lifespans of 4.17±0.31 days and 4.53±0.3 days, respectively, compared to that for nematodes feeding on the *E*. *coli* OP50 strain (Fig 2d and 2e). While almost all target bacteria reduced the lifespan of worms, *E*. *coli* O145:NM B485 83–75 (E14) and *E*. *coli* O145:NM B486 14728(E15) increased the survival rate of the worms. This result indicates that most STEC strains used in the study significantly reduce the longevity (lifespan) of *C*. *elegans* wild type N2.

### Heat-treated STECin the *C*. *elegans* killing assay

**Fig 3:**
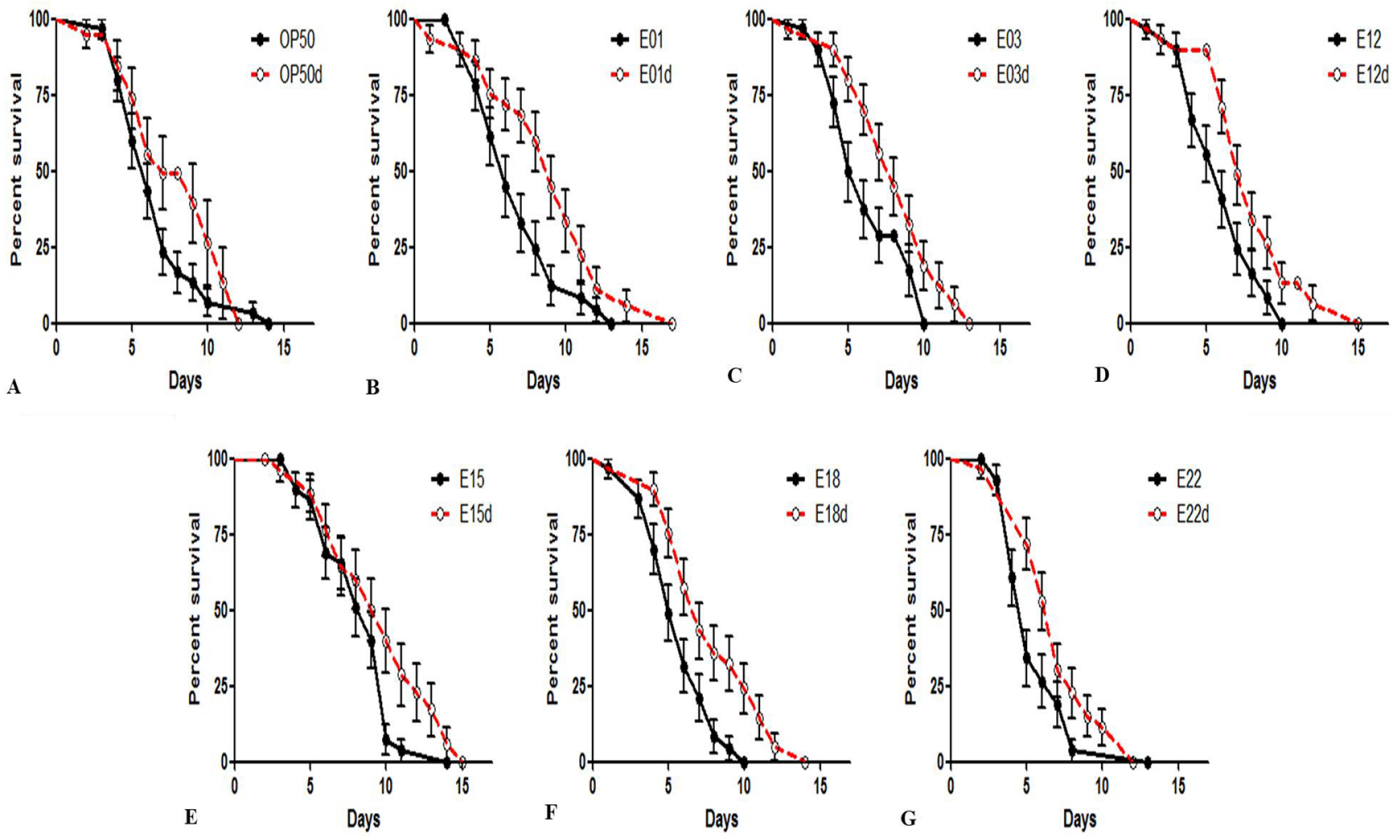
Toxicity of heat-treated and live strains in the *C*. *elegans* model. A) Heat-killed *E*. *coli* OP50 compared to live *E*. *coli* OP50. B) Heat-killed E1: O104 compared to live E1: O104. C) Heat-killed E3: O111 compared to live E3: O111. D) Heat-killed E12: O121 compared to live E12: O121. E) Heat-killed E15: O145 compared to live E15: O145. F) Heat-killed E18: O145 compared to live E18: O145. G) Heat-killed E22: O157 compared to live E22: O157. The survival curves of nematodes feeding on both heat-killed STEC strains and the *E*. *coli* OP50 strain were examined. The live strains were compared to the heat-killed strains. All heat-treated bacterial strains used in this assay increased the lifespan of *C*. *elegans* when compared to the control strains. This suggests that the toxic factors of STEC strains are heat-labile.

In order to determine the difference between heat-killed bacteria and live bacteria, we determined the lifespan of *C*. *elegans* fed heat-killed STEC strains (pellet). The effect of heat-killed STEC (80°C, 10min) strains in N2nematodes was quantitatively assessed at 20°C. It was determined that all of the heat-treated STEC strains significantly increased the survival rate of *C*. *elegans* compared to that of the non-heat-killed STEC strains. With respect to the control *E*. *coli* OP50 strain, these results revealed that the N2 worms fed heat-killed STEC (E1, 3, 12, 15, 18, 22) bacterial strains and the control *E*. *coli* OP50 strain had significantly longer lifespans (Fig 3). In contrast, a shorter lifespan was observed for *C*. *elegans* that were fed the non-heat-treated (live) *E*. *coli* bacterial strains. As shown in Fig 3a, the heat-killed *E*. *coli* OP50 strain increased the longevity of the worms (7.81±6.57 days) compared to the live E. coli OP50 strain. Likewise, the heat-treated bacteriaE1 (8.78±0.77 days), E3 (8.01±0.53 days), E12 (7.85±0.60 days), E15 (9.4 6 ± 0.68 days), E18 (7.72±0.58 days) and E22 (7.05±0.45 days) improved the survival rate of *C*. *elegans* compared to the live STEC strains E1 (6.78 ±0.51 days), E3 (6.21 ± 0.47 days), E12 (5.95 ± 0.42 days), E15 (8.20 ± 0.45 days), E18 (5.64 ± 0.38 days) and E22 (5.52 ± 0.41 days). These results suggest that heat treatment at 80°C causes the STEC strains to lose their toxin effect when compared to the live STEC strains in the *C*. *elegans model*.

### Identification of the stx1 and stx2 genes in STEC strains

**Fig 4:**
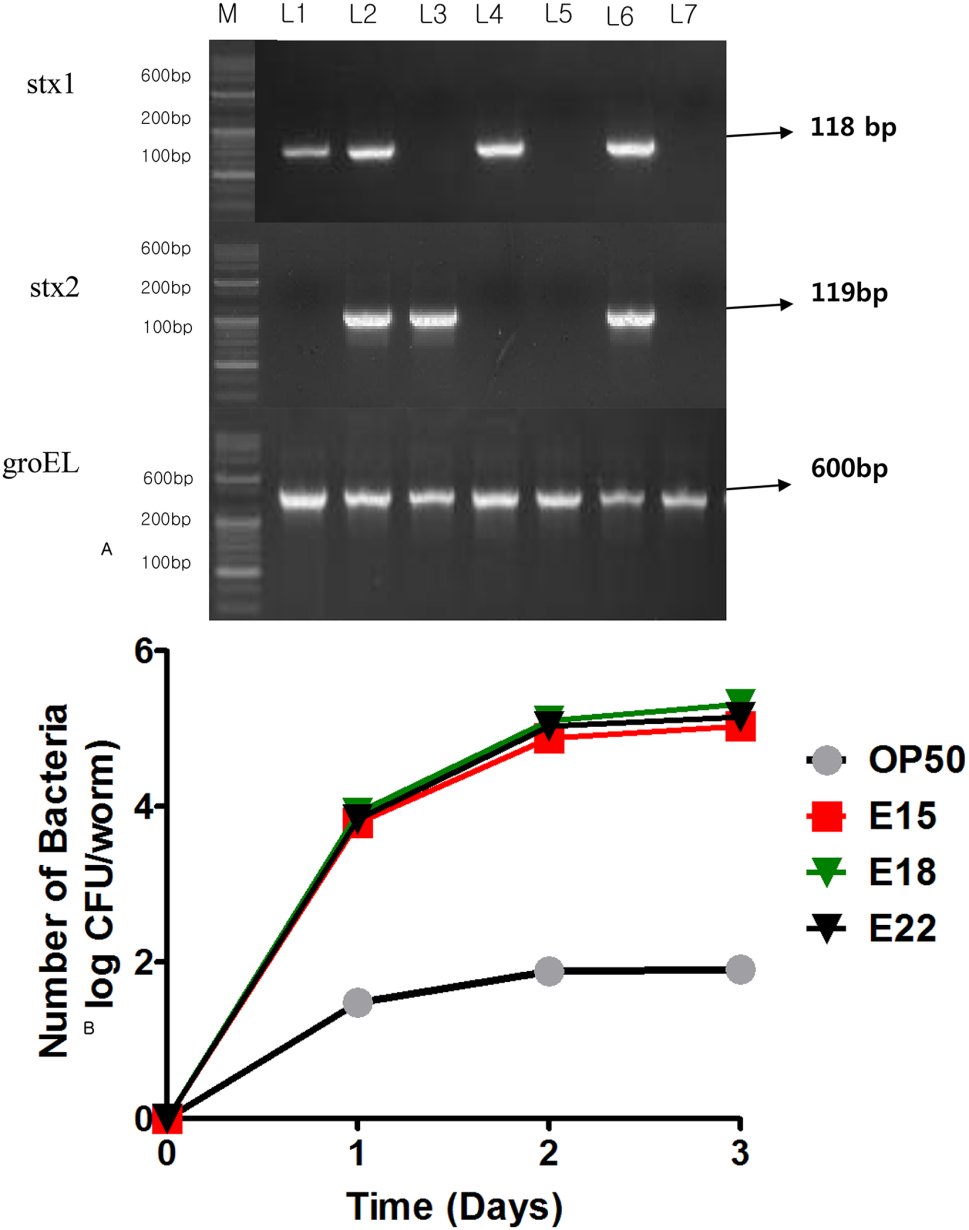
Conventional PCR and colonisation assay. **Conventional PCR**: Based on the results of the life span assay, 6 STEC strains (L1—E1; L2—E3; L3—E12; L4—E15; L5—E18; L6—E22; L7 –OP50) out of the 24 strains were selected for further testing for the presence of toxin genes (stx1, stx2 and groEL) using 3 primer pairs (STX1-F/R-O157; STX2-F/R-O157; GroEL-F/R-O157) targeting the genes specific for toxin production(stx1, stx2 and groEL). **Colonisation assay**: The STEC strains colonised in the intestinal tract of L2 worms (log CFU/worm). p<0.001 was obtained for comparison with the *E*. *coli* OP50 control group by the t-test. The total numbers of worms (*C*. *elegans*) tested in each group are indicated by n.

Conventional PCR was performed to confirm that STEC strains (L1—E1; L2—E3; L3—E12; L4—E15; L5—E18; L6—E22; L7 –OP50) produce stx1 and stx2 toxins. Based on our results, E1, E3 and E22 reduced the brood size and the lifespan of *C*. *elegans*. Compared to *E*. *coli* OP50, which possesses the stx1 gene, E12 and E18 lack the stx1 gene. In addition, E15, which reduced the progeny and affected the survival rate of the worms, possessed the stx1 gene (Table 1). Based on the PCR assay, the STEC toxin gene was confirmed by the detection of virulence genes (stx1 and stx2) in addition to groEL. The 3 primer pairs (Table 2) used in the assay do not interfere with each other and generate amplification products 118, 119, and 600bp in size (Fig 4a).

**Table 1:** Bacterial (*E*. *coli)* strains used in the study and their respective sources.

Arbitrary	Isolates^a^						
	ID No.^b^	Original ID no.	Serotype	Source(reference)	*stx1*	*stx2*	*groEL*
**OP50**		*E*. *coli* OP50			-	-	+
**E1**	B471	1.2673	O104:H12	Co	+	-	+
**E2**	B472	JB1-95	O111:H-	H	NT	NT	NT
**E3**	B473	96–3166	O111:NM	H	+	+	+
**E4**	B475	TB226	O111:NM	H	NT	NT	NT
**E5**	B476	8361	O111:H8	H	NT	NT	NT
**E6**	B477	12893	O111:H8	H	NT	NT	NT
**E7**	B478	14895	O111:H8	H	NT	NT	NT
**E8**	B479	DA-1	O121:NM	H	NT	NT	NT
**E9**	B480	97–3068	O121:H19	H	NT	NT	NT
**E10**	B481	03–4064	O121:NM	H	NT	NT	NT
**E11**	B482	11435	O121:H19	H	NT	NT	NT
**E12**	B483	9918	O121:NM	H	-	+	+
**E13**	B484	10896	O121:NM	H	NT	NT	NT
**E14**	B485	83–75	O145:NM	H	NT	NT	NT
**E15**	B486	14728	O145:NM	H	+	-	+
**E16**	B487	940941	O145:H-	H	NT	NT	NT
**E17**	B488	6383	O145:NM	H	NT	NT	NT
**E18**	B489	BCL73	O145:NM	Co	-	-	+
**E19**	B490	8235	O145:NM	H	NT	NT	NT
**E20**	B491	6896	O145:NM	H	NT	NT	NT
**E21**	B492	sakai	O157:H7	H	NT	NT	NT
**E22**	B493	C7927	O157:H7	H	+	+	+
**E23**	B494	FSIS413-95	O157:H7	Gb	NT	NT	NT
**E24**	B495	380–94	O157:H7	H	NT	NT	NT

Bacterial strains used in this study and specification of three primers based on conventional PCR assay **(**H- Human; Gb- ground beef; Co- cow, NT- Not Tested)

**Table 2:** Oligonucleotide primer sequences used in this study with respect to product size.

Targeting genes	Primers	Primer Sequence (5’→3’)	Product Size(bp)	Annealing Temperature (°C)	Source
**stx1**	STX1-F-O157	GAAAGCGATGCAGCTATTA	789	60	Chou et al., 2013
	STX1-R-O157	GGATAATTTGTTTGCAGTTG			
**stx2**	STX2-F-O157	TATTATTTAAATGGGTACTGTGC	1073	60	
	STX2-R-O157	ATGTGTCATCCTCATTATACTTG			
**groEL**	GroEL-F-O157	CCGTAACGTAGTTCTGGATA	1493	60	
	GroEL-R-O157	CTAAGTCAGCTGCATCGTT			

### Larval population density predetermines adult lifespan

**Fig 5:**
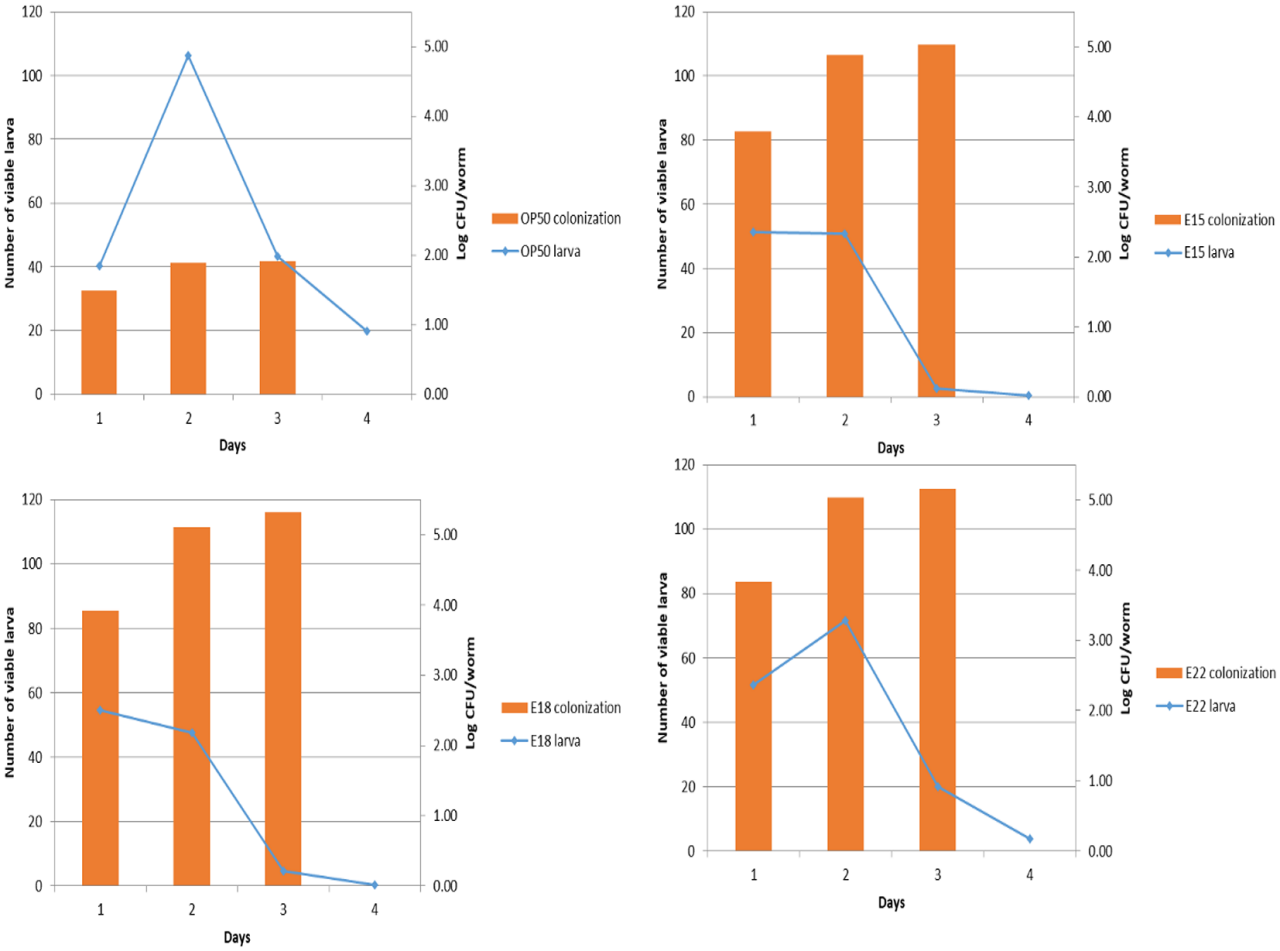
Correlation between the life span and fertility of *C*. *elegans* infected with STEC strains. The STEC (E15, E18 and E22) strains showed an increase in colonisation, which correlates with a decrease in longevity and fertility of the worm (*C*. *elegans*). This is considered evidence of STEC pathogenicity in the *C*. *elegans* model.

Similar to the pathogenic colony formation in the human gastrointestinal tract, we observed the colony formation of STEC strains in the intestine of *C*. *elegans*. These results indicated that the intestines of the worms possessed a high colony number (3.34 and 4.70 log CFU/worm on each day) of E22 on the first and third days. In contrast, there were 3.23 and 2.40 log CFU/worm of E18 and E15, respectively, on the first day and subsequently 4.23 and 4.12 log CFU/worm of E18 and E15, respectively, on the third day of growth (Fig 4b). However, *C*. *elegans* was colonised with 1.92 and 3.59 log CFU/worm of the control *E*. *coli* OP50 strain on each day. Hence, the tested STEC strains (E15, E18 and E22) colonised to a greater degree in *C*. *elegans* compared to the *E*. *coli* OP50 strain. This result correlates with the reduction in the life span of *C*.*elegans*. STEC colonisation is proportional to a decrease in the longevity and fertility of the worm (Fig 5). In particular, E22 was observed to have accumulated to more than 1 log CFU/worm in all days compared to the control *E*. *coli* OP50 strain.

## Discussion

The *C*. *elegans* model has been used to determine the pathogenesis and host susceptibility to various pathogens [37]. Specifically, bacterial virulence and host susceptibility mechanisms identified in *C*. *elegans* are often well preserved. Thus, many bacterial mutants that are avirulent in *C*. *elegans* are also avirulent in other mammals [38]. Moreover, a variety of pathogens with specific modes of infection were analysed using the *C*. *elegans* model.

Previous studies have utilized *C*. *elegans* as an in vivo model to study the molecular mechanism regarding the virulence of gene-based pathogens of bacterial species. Previous studies reported on the toxic effects and pathogenicity of other enteropathogenic group (EEPC) strains with virulence factor expression and inflammatory responses in *C*. *elegans* [39]. Indole-based toxicity in *C*. *elegans* is likely to be protective in mammals because they suppress the expression of bacterial virulence genes and shiga toxin at high concentrations [40]. Moreover, our findings suggest that STEC strains possessing the toxin-producing gene [41–42] reduce the life span of *C*. *elegans* (Fig 3). Likewise, most of the *Shigella* toxins have been found to be exotoxins [43–44], and they belong to a small molecular protein family such as peptides and have been reported to be heat sensitive [45]. Our results highlight the utility of *C*. *elegans*-pathogen systems in conjunction with heat-treated *E*. *coli*, which showed reduced toxicity. Indeed, these methodologies may be broadly applicable in identifying the relationship between host-toxin interaction and the characterization of the toxin properties in many organisms [5]. Most food poisoning is caused by the toxicity of non-*E*. *coli* O157 strains as well as *E*. *coli* O157:H7. Although many studies have reported on the toxicity of *E*. *coli* O157:H7, there is little research on the toxicity of non-O157 strains. Therefore, we identified the virulence of pathogenic *E*. *coli*, including O157 strains and non-O157 strains, using *C*. *elegans* as a model system.

There have been few studies reported previously on the interaction between pathogenic *E*. *coli* and *C*. *elegans* [46]. Our results demonstrate that STEC strains infect and kill nematodes. We showed that STEC strains decrease the brood size and lifespan of *C*. *elegans*. We also demonstrated that pathogenic *E*. *coli* strains form colonies and increase in population in the intestinal tract of worms. Moreover, our genetic analysis and heat-killed bacteria killing assay results associatedstx1 and stx2 of STEC strains with its toxicity. Our results suggest that *C*. *elegans* is a suitable alternative model for studying the infection of pathogenic bacteria, including STEC strains, in hosts in vivo.

These results suggest that *C*. *elegans* is highly susceptible to infection with most of the STEC strains, and the normal morphology of the intestine in the infected animals may be affected. The fertility and lifespan assay showed that most of the STEC strains reduced the brood size and life span of *C*. *elegans* similar to that observed for *C*. *elegans* infected with *E*. *coli*. Each of the E1, E3, E12, E18 and E22STEC strain serotypes was selected for screening for STEC strain virulence in *C*. *elegans* because they were the most toxic of the serotypes. In addition, E15, which was associated with a survival rate similar to that of *E*. *coli* OP50, was used in further experiments. Due to certain limitations in the nematode model, the worm model may not predict a 100% correlation[5]. However, infection can play a major role in the ability of human pathogens to interact with a host, the mode of virulence, and the sensitivity that can be expressed in *C*. *elegans* [47–48]. As such, it is quite possible that the clinical isolates, which were non-pathogenic to *C*. *elegans* in our assay, did not express the appropriate virulence factors. Consequently, the differences in pathogenicity for some of the clinical isolates in this study may be due to differences in the infection conditions. This observation raises fascinating questions concerning how the expression of various genes differs depending on the infection conditions (Fig 2).

Based on the results obtained, it had been determined that the STEC strains are responsible for reducing the brood size and lifespan of L2 worms. This may be due to the colonisation of virulent bacteria. It was also reported that the *E*. *coli* O157 strain causes toxicity based on colony formation in the gastrointestinal tract of humans. Hence, STEC strains may colonize in the intestine of the nematodes and cause toxicity towards *C*. *elegans*. Thus, *C*. *elegans* can be used as a model system to screen virulence mechanisms of STEC strains when compared to the control *E*. *coli* OP50 strain.

The *E*.*coli* O157:H7 strain, the most common type of STEC, harboursa virulence plasmid that possesses genes such as stx1, stx2, eae, and ehxA [49–51], which are more commonly associated with severe human diseases[52–53]. It can produce two different shiga-like toxins, stx1 and stx2, which are encoded on prophages embedded in the genome. The stx1 is very similar to the shiga toxin of *Shigella dysenteriae*, and stx2 is genetically and immunologically distinct from stx1 [54]. Also, a recent report suggested that stx1 of EHEC is almost entirely cell-associated and causes many diseases in humans in vivo only when it is released by bacterial lysis, whereas stx2 can be released by two different mechanisms from bacterial cells into the extracellular milieu [55]. Our PCR and electrophoresis results showed that E1, E3, E15 and E22 possessed the stx1 gene, while E12 and E18 exhibited strong virulence towards *C*. *elegans* via stx2(Table 1). As previously reported, *E*. *coli* O157:H7 possessed stx1 and stx2, and the heat-treated *E*. *coli* O157:H7 strain did not cause an early death of *C*. *elegans* in the heat-killed bacteria killing assay. It has been reported that the enzyme activity of stx1 is greatly decreased at higher temperatures (Brigotti et al., 2004), whereas stx2 is heat-stable and not inactivated at higher temperatures (Rasooly and Do, 2010). As a result, all of the heat-treated STEC strains significantly increased the lifespan of *C*. *elegans* compared to control STEC strains, as suggested by a significantly longer longevity of the L2 worms fed heat-killed target bacteria and the control *E*. *coli*OP50 strain (Fig 3). The most prominent virulence genes evaluated in the present study were significant in that they were detected at a much higher rate pathogenetically (Fig 4a), and Table 1shows the adhesion-encoding genes focG, focA, and sfaD that encode a receptor with an auto-transporter protein that were typically associated with extra-intestinal infections. In addition, theywere required for synthesis of the F1C pilus and were upregulated during a urinary tract infection (UTI) caused by the *E*. *coli* strain CFT073 [5]. It is imperative to note that the genes themselves may not be directly involved in infection, but they could represent the effect of a combination of genes interlinked with the selective virulence genes of pathogenicity [21] that were not screened for during this PCR analysis. Moreover, regulatory genes required for the mRNA expression of virulence genes may be suppressive or not present, which results in the dissimilarity between the non-virulence type and pathogenicity in the *C*. *elegans* assay [46].

In conclusion, the virulence factor of the *E*. *coli* O157 strain is primarily shiga toxin 1, and the heat-killed *E*. *coli* O157 strain could not colonize in the intestine of *C*. *elegans* and cause toxicity due to an inactivated stx1. Although some non-O157 strains are believed to be similar to the O157 strain, both E12 and E18 are considered to be toxic by forming colonies in the intestinal tract of *C*. *elegans* rather than producing shiga toxins. The relatively rapid method of conventional PCR was used to identify both the stx1 and the stx2 genes and confirm the presence of STEC. Using *C*. *elegans* as an invivo model for the study of the virulence of shiga toxin-producing *E*. *coli*, our work further focused on detecting toxin genes with similar PCR profiles in patients within a short period of time and may provide the first evidence of a link between cases consistent with common-source outbreaks. The PCR analysis is a valuable diagnostic and epidemiological tool, which facilitates the analysis of STEC before it causes severe disease in humans.

## Material and methods

### Bacterial strains and growth media

The STEC strains used in this study are listed in Table 1. The US Food Fermentation Laboratory Culture Collection (Raleigh, NC, USA) and the Agricultural Research Service, Eastern Regional Research Center (Wyndmoor, PA, USA) provided4 STEC *E*. *coli* O157:H7 strains and 20non-O157 STEC strains, consisting of the 4 serogroups O104, O111, O121,and O145 from various sources such as cow, ground beef, and humans. The bacterial strains were stored at –80 °C in tryptic soy broth (TSB, BD Biosciences, San Jose, CA, USA) supplemented with 30% glycerol. Each culture was streaked from frozen stocks onto tryptic soy agar (TSA, BD Bioscience) and incubated at 37 °C for 24 h.

### Population density-dependent egg-laying assays

#### Nematode and stage of development

*C*. *elegans* is studied in four stages (L1, L2, L3 and L4). The stage immediately following egg hatching is called the L1 stage. Adult worms lay their eggs, and the stage before *C*. *elegans* lays eggs is called the L4 stage. It is maintained on 35-mm diameter NGM (Nematode Growing Media) agar plates seeded with 50μL of *E*. *coli* OP50, and it is stored at 20°C. *C*. *elegans* (N2) strains were obtained from the *Caenorhabditis* Genetic Center (CGC; MN).

#### Nematode synchronizing

The young adult worms hatched from eggs were treated with sterile water containing 0.5 M NaOH in addition to 0.5% bleaching solution (sodium hypochlorite and sodium hydroxide) for synchronization. Briefly, the assay was performed as previously described [56]. After vortexing in 2-min intervals, the worms were washed with M9 buffer via centrifugation (1200 × *g* for 200 sec) and resuspended, which was subsequently repeated three times. To prepare worms for all assays, approximately 3000 of the synchronized eggs were hatched in M9 buffer at 20°C overnight. The L1 larvae were subsequently transferred to NGM agar containing a lawn of *E*. *coli* OP50 and incubated at 25°C for 48 h to reach the L4 stage.

#### *C*. *elegans*fertility assay

L2 young adult worms were transferred to new NGM plates containing 50 μL of the target bacterial strain cultured in TSB every day until they did not produce progeny. The NGM plates containing eggs were incubated at 20°C for another 48 h, and the number of progeny was counted for each adult worm tested at the end of 3 days. The fertility of worms was monitored for wild-type L2 [57].

### Lifespan assay

The longevity of *C*. *elegans* was determined by a previously described method [58]. In the experiment, 50 μL of the target bacterial strain cultured in TSB at 25°C for 24 h was spread on a 35-mm diameter NGM agar plate and incubated overnight at 37°C. Each plate was seeded with 30 *C*. *elegans* L4 stage larva or young adults grown on *E*. *coli* OP50. All plates were incubated at 25°C, and dead worms were counted every 24 h. Liveworms were transferred onto fresh NGM agar plates seeded with the target bacterial strain every 2 days. The experiment was performed in triplicate for each strain. Each plate was examined until the worms died. In order to compare the effects of the *E*. *coli* (STEC) strains, worms were grown on *E*. *coli* OP50, and the mean life span (MLS) was calculated by using the formula previously described [59].

### Effect of heat on toxic compounds

#### Heat-killed bacteria killing assay

The assay plates were prepared as follows using the procedures outlined above for the killing assay. The STEC strains and the *E*. *coli* OP50strain were incubated overnight at 37°C in TSB medium. Using a dry bath (Vision Scientific -251D12), the bacteria were treated aseptically at 80°C for 10 min, and 50-ml aliquots were spread-plated on NGM agar as well as Eosin Methylene Blue (EMB) agar (selective media for *E*. *coli*) to identify whether the bacteria were killed by heat-treatment.

#### Colonisation-virulence interaction

The number of colonised worms and their bacterial load were estimated in individual progeny samples at day 3 in parallel with the number of bacteria present on the plate. Immediately following the killing assays, bacterial colonisation in the intestine of *C*. *elegans* was quantified by the spread-plating method to determine the number of live bacteria in the worm (L2). The young adult L4 stage worms were fed either STEC strains or *E*. *coli* OP50 for 3 days at 20°C. Five L2 worms were picked after 1 day and 3days. The surface bacteria of *C*. *elegans* were removed by washing the worms subsequently three times in 1ml of 1% Triton X-100, and finally,5 worms were disrupted using a pestle with 20μL of 1%Triton X-100. They were diluted to 10^−1^, 10^−2^, 10^−3^ and 10^−4^ with 1ml of M9 buffer and then plated on EMB agar. Bacterial colonies (log CFU/ml) were counted after 16 h of incubation at 37°C. Each experiment was performed on the 1^st^ and 3^rd^ days.

### Evaluation of potential virulent genes associated with pathogenesis

Primary identification and characterization of the stx1 and stx2virulent genes in STEC responsible for toxin production were performed using conventional PCR with the primers given in Table 2. The primary PCR results for stx1, stx2, and groEL in all strains are included in the study. For the PCR, the bacterial strains were grown overnight on TS agar. One of the bacterial colonies was suspended in 100 μL of lysis buffer (50 mMKCl,10 mM Tris-HCl, pH 8.3,2.5 mM MgCl^2^,0.45% NP-40, 0.45% Tween 20) and 100 μLof Tris-EDTA (TE) buffer solution (pH 7.4) and boiled for 15 min at 95°C. After centrifugation at 13,000 rpm for 1 min, the supernatant was used directly in the PCR using standardized reaction conditions (denaturation at 95°C for 10 min; 35 cycles of 95°C for 15 sec, 60°C for 30 sec, and 72°C for 30 sec; and a 3-min final extension at 72°C).

### Statistics

The reproducibility of the qPCR was determined using three independent experiments conducted in triplicate. The data obtained from each experiment were expressed as the mean, and the standard deviation is shown as error bars. The asterisk denotes p<0.001 compared to the *E*. *coli* OP50 control group calculated by the t-test. The total numbers of samples in each group are indicated by n (Fig 4b). Differences in the survival of *C*. *elegans* in the infection assays were determined using Graph Pad Prism version 5.00 (www.graphpad.com).

## Supporting information

S1 Fig Outline of the manuscript—Host fertility and lifespan affected by shiga toxin-producing *Escherichia coli* (STEC) in a *Caenorhabditis elegans* model. (PDF)
