# Supplementary material for: In vivo screening platform for shiga toxin-producing Escherichia coli (STEC) using Caenorhabditis elegans as a model
Source: PLoS One. 2018 Feb 28;13(2):e0193277. doi: 10.1371/journal.pone.0193277 (PMC5831388; doi:10.1371/journal.pone.0193277)
Supplement: S1 Fig — (PDF) [file pone.0193277.s001.pdf]

Screening the effect of EHEC and Shiga toxin-producing *Escherichia coli* (STEC) on fertility and lifespan of *Caenorhabditis elegans*

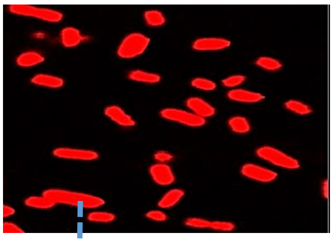

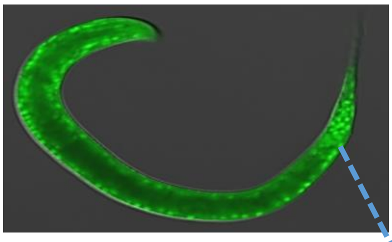

***Escherichia coli* strains used in the study**

E. coli – OP50 as control (1)

E. coli – STEC strains (24)

Total n = 25

STEP- 1

Pathogens shiga toxin producing *E.coli*

Cultivated in TSB Media

Source of the Strains applied in the study

U.S. Food Fermentation Laboratory Culture Collection (Wyndmoor, Pa., U.S.A.)

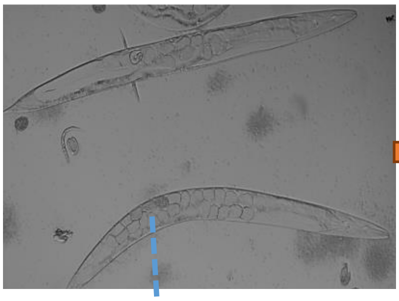

Nematode (*Caenorhabditis elegans*)

Applied as an In-vivo Model

STEP- 3

Nematode synchronizing Wild Type (N2)

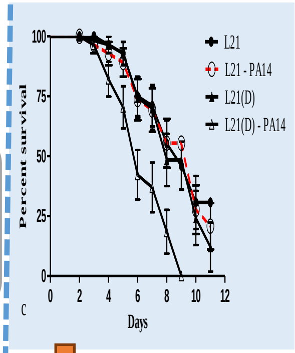

STEP- 4

Fertility assay

STEP- 5

Lifespan assay

STEP- 7

Heat-killed STEC *E. coli* in *C. elegans* - killing assay

80°C for 10 min

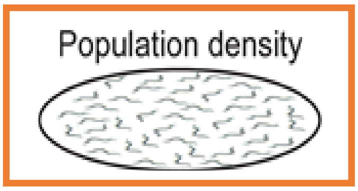

Based on the Colonization-virulence interaction of STEC strains in the intestine of the N2

The STEC *E. coli* (E15, E18 and E22) strains showed increase in colonization which is perpendicularly correlated with decrease in longevity and fertility of the worm (*C. elegans*).

Based on the Colonization-virulence interaction of STEC strains in the intestine of the worms

Most of N2 animals feeding on STEC plates were significantly short-lived compared to those on *E. coli* OP50 plates. (a) *E. coli* O101 strains, (b) *E. coli* O111 strains, (c) *E. coli* O121 strains, (d) *E. coli* O145 strains and (e) *E. coli* O157strains.

Conventional and qPCR are rapid and accurate detection methods standardized for **TARGETING GENES stx1 and stx2**

PRIMER USED IN THE STUDY

STX1-F/R-O157 – 789bp

STX2-F/R-O157 – 1073bp

GroEL-F/R-O157 – 1493bp

STEP- 8

All heat-treated bacteria strains used in the assays increased the longevity against original strains.

Heat Liable Toxic Compounds

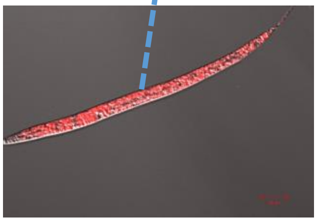

Conclusion

Among 24 STEC strains E12 and E18 are considered to be toxic by forming colony in the intestinal tract of *C. elegans* based on the shiga toxin in comparison with the control OP50 Strain

FUTURE APPLICATIONS

1. Detection of toxin genes with similar PCR profile in patient
2. Study on Mechanism based on Host Pathogen Interaction
